# Rescue of corpus callosum agenesis in two ciliary mutants by the short repressor isoform of Gli3

**DOI:** 10.1186/2046-2530-4-S1-P36

**Published:** 2015-07-13

**Authors:** C Laclef, I Anselme, L Besse, M Catala, D Baas, M Paschaki, B Durand, S Schneider-Maunoury

**Affiliations:** 1 Sorbonne Université, UPMC Univ. Paris 6, UMR7622, F-75005 Paris, France; 2 CNRS, INSB, UMR7622, F-75005 Paris, France; 3 Université Lyon 1, UMR5534, F-69622 Villeurbanne, France; 4 CNRS, CGPMC-UMR5534, UMR5534, F-69622 Villeurbanne, France

## 

Agenesis of the Corpus callosum (AgCC) is a frequent brain disorder found in over 50 human congenital syndromes including ciliopathies. Here, we report a severe AgCC in Ftm/Rpgrip1l knock-out mice, which provide a valuable model for Meckel-Grüber syndrome. Ftm encodes a protein of the ciliary transition zone, which is essential for ciliogenesis in some, but not all cell types in mice, including neuroepithelial cells in the developing forebrain.

We show that AgCC in Ftm^-/- ^fetuses results from a mislocalization of guidepost cells in the dorsomedial telencephalon and not from a reduction of callosal neurons in the cortex. This abnormal distribution of the guideposts primarily results from patterning defects in the medial telencephalon, which relies on Gli3 processing defects. These patterning defects, mispositioning of dorsomedial guideposts and AgCC can all be rescued by reintroducing Gli3R in Ftm^-/- ^embryos, provided by the Gli3D699 allele, which produces only the short repressor isoform of Gli3. This rescue of AgCC of Ftm^-/- ^mutants by one allele of Gli3D699 confirms and completes our knowledge on the pre-eminent role of Gli3 in CC formation.

Furthermore, Gli3D699 also rescue AgCC in Rfx3^-/- ^mutant, another ciliary mutant deficient for a transcription factor controlling several ciliary genes. Rescuing the CC formation in two independent ciliary mutants by a single molecule, namely Gli3R, highlight the crucial role of primary cilia in maintaining the proper level of Gli3R required for CC morphogenesis. These data demonstrate that Gli3 processing is the major outcome of primary cilia function in CC formation.

